# Clinical experience with eravacycline-based combination therapy for carbapenem-resistant *Acinetobacter baumannii* pneumonia: a prospective observational case series

**DOI:** 10.3389/fphar.2026.1796494

**Published:** 2026-05-07

**Authors:** Ying Xiao, Yu Cheng, Xiaohong Xu, Hongqiang Qiu, Xuanxi Huang, Zhiqiang Xue, Meili Cai, Qing Wang, Yanmei Guo, Hui Zhang

**Affiliations:** 1 Department of Critical Care Medicine, Fujian Medical University Union Hospital, Fuzhou, China; 2 Department of Pharmacy, Fujian Medical University Union Hospital, Fuzhou, China; 3 Department of Laboratory Medicine, Fujian Medical University Union Hospital, Fuzhou, China

**Keywords:** *Acinetobacte*r baumannii, carbapenem resistance, clinical outcomes, combination therapy, eravacycline, pharmacokinetics

## Abstract

**Objective:**

This study was conducted to investigate the clinical response, safety, and pharmacokinetic (PK) profile of eravacycline-based combination therapies in patients with pulmonary infections caused by carbapenem-resistant *Acinetobacter baumannii* (CRAB).

**Methods:**

A prospective observational case series was conducted on patients with CRAB pneumonia treated at Fujian Medical University Union Hospital between February 2024 and August 2025. Prior to treatment, eravacycline minimum inhibitory concentration were determined. Patients received eravacycline-based combination therapy according to their susceptibility results. Data on clinical responses, microbiological clearance, adverse events, and 28-day all-cause mortality were collected. Eravacycline blood concentrations were measured in a subset of patients.

**Results:**

Twenty-two patients with CRAB pneumonia (21 with ventilator-associated pneumonia) were enrolled; all had a Clinical Pulmonary Infection Score >6. The mean Acute Physiology and Chronic Health Evaluation II (APACHE II) scores and the Sequential Organ Failure Assessment (SOFA) scores were 13.77 and 6.23, respectively. All CRAB isolates were susceptible to eravacycline (ChiCAST breakpoint ≤1 mg/L). The clinical response rate, microbiological clearance rate, and 28-day mortality were 72.7%, 86.4%, and 13.6%, respectively. Two patients exhibited hypofibrinogenemia, considered an adverse drug reaction related to eravacycline. No associated bleeding events or other adverse events were observed. Nonresponders had significantly higher SOFA and APACHE II scores than clinical responders. Eravacycline PK was assessed in 14 patients and showed considerable interindividual variability. Among the PK parameters, C_max_ correlated significantly with clinical response (*r* = 0.729, *p* = 0.003).

**Conclusion:**

The preliminary findings of this prospective observational case series indicate that individualized eravacycline-based combination regimens are associated with promising clinical outcomes and microbiological clearance, with good tolerability and a low incidence of adverse events. Additionally, initial blood concentration data provide a reference for future exploration of PK/PD targets in CRAB pneumonia. However, due to the small sample size and limited statistical power, these findings should be considered preliminary and require validation in larger prospective cohorts.

## 1 Background

*Acinetobacter baumannii*, an opportunistic Gram-negative *bacillus*, can survive under diverse environmental conditions. It is a prevalent nosocomial pathogen that can cause infections across multiple organ systems, with the lungs being the most commonly affected site in hospital-acquired infections (HAPs) (Kubin et al., 2025). As a result, *Acinetobacter baumannii* is a primary pathogen in hospital-acquired pneumonia, especially ventilator-associated pneumonia (VAP). However, *A. baumannii* exhibits intrinsic resistance to conventional antibiotics and demonstrates high resistance to carbapenems, leading to the emergence of carbapenem-resistant *A. baumannii* (CRAB) (Peleg et al., 2008). According to China Antimicrobial Surveillance Network (CHINET) data, the resistance rates of *A. baumannii* to imipenem and meropenem in China have increased from 31.0% to 39.0% in 2005 to 64.5% and 64.7% in 2025, respectively (Yan et al., 2025), and have remained consistently high over the past 5 years. Once resistance develops, CRAB infections become extremely difficult to treat, as few effective therapeutic options are available in clinical practice. To promote the advancement of effective antimicrobial therapies, the World Health Organization (WHO) designated CRAB as a “critical priority” pathogen on its 2024 priority list of antibiotic-resistant bacteria (Sati et al., 2025). Furthermore, the U.S. Centers for Disease Control and Prevention (CDC) recently upheld its classification of CRAB as an “urgent threat” (Prevention, 2025).

Eravacycline is a novel, fully synthetic fluorocycline antibiotic characterized by a broad spectrum of antimicrobial activity. This antibiotic demonstrates strong efficacy against clinically important multidrug-resistant pathogens, including carbapenem-resistant *Enterobacterales* (CRE), CRAB, methicillin-resistant *Staphylococcus aureus* (MRSA), and vancomycin-resistant *Enterococcus* (VRE) (Alosaimy et al., 2020a). The mechanism of action entails binding to the 30S ribosomal subunit, thereby inhibiting bacterial protein synthesis. Research has indicated that the minimum inhibitory concentration (MIC) of eravacycline against CRAB is between two- and eight-fold lower than that of tigecycline (Zhao et al., 2019; Zou et al., 2023). In 2022, the European Society of Clinical Microbiology and Infectious Diseases (ESCMID) included eravacycline among novel antimicrobial agents with potential to treat carbapenem-resistant organisms (CROs) (Paul et al., 2022). Similarly, the 2024 Infectious Diseases Society of America (IDSA) guidance for the treatment of antimicrobial-resistant infections recommended eravacycline as a therapeutic alternative for infections caused by CRE and CRAB (Tamma et al., 2024). The “Guidelines for the Diagnosis, Treatment, Prevention, and Control of Infections Caused by Carbapenem-Resistant Gram-Negative Bacilli,” published by Chinese experts in February 2023, emphasize that eravacycline attains higher pulmonary tissue concentrations and is associated with a reduced incidence of adverse events relative to tigecycline in the management of pneumonia caused by CRAB (Zeng et al., 2023). A retrospective observational study conducted in the United States included 46 patients diagnosed with CRAB infections who underwent eravacycline therapy for more than 72 h. The findings indicated that eravacycline demonstrated both efficacy and a favorable tolerability profile. Notably, only one patient reported a potentially drug-related adverse event, characterized as Grade 1 diarrhea, which did not necessitate treatment cessation (Alosaimy et al., 2022).

Since its approval in China in 2023, eravacycline has been increasingly used in clinical settings, particularly for the treatment of HAPs caused by multidrug-resistant pathogens. However, the current body of evidence regarding its efficacy, especially when used in combination regimens for CRAB pneumonia, remains insufficient. To address this knowledge gap, the present prospective case series was conducted to evaluate clinical responses, microbiological eradication rates, adverse events, and 28-day all-cause mortality among patients with CRAB pulmonary infections treated with eravacycline-based combination therapies. Additionally, plasma concentrations of eravacycline were assessed to delineate its PK profile and investigate potential associations with therapeutic outcomes, thereby generating clinically pertinent data to guide the management of CRAB pneumonia.

## 2 Materials and methods

### 2.1 Study design

This prospective study enrolled patients diagnosed with pulmonary infections caused by CRAB. The participants were treated with a combination antimicrobial regimen centered on eravacycline, according to bacterial culture and antimicrobial susceptibility testing results. Clinical data were collected to evaluate the clinical response rate, microbiological eradication rate, incidence of adverse events (AEs), and 28-day all-cause mortality. Additionally, plasma concentrations of eravacycline were determined.

#### 2.1.1 General information

Patients diagnosed with pulmonary infections caused by CRAB treated at Fujian Medical University Union Hospital in Fuzhou, Fujian Province, China, were prospectively recruited from February 2024 to August 2025.

The inclusion criteria were as follows: Patients aged 18 years or older, admitted within the specified period, and diagnosed with HAP or VAP according to the Chinese Thoracic Society guidelines. Diagnosis required the presence of new or progressive infiltrates on chest imaging in conjunction with at least two of the following clinical features: fever exceeding 38 °C, purulent respiratory secretions, or leukocytosis/leukopenia. Additionally, patients were required to have CRAB as a confirmed or predominant pathogen (a bacterial load of grade ≥ +++ in respiratory specimens), with susceptibility to eravacycline.

The exclusion criteria were as follows: patients who were pregnant or breastfeeding, had concurrent urinary tract infections or bacterial infections of the central nervous system, were diagnosed with a terminal illness, had a documented hypersensitivity to tetracycline-class antibiotics, or had received eravacycline treatment for fewer than 4 days.

#### 2.1.2 Microbiological culture, identification, MIC determination, and antibiotic resistance gene sequencing

According to the 2024 edition of the Clinical and Laboratory Standards Institute (CLSI) guidelines (CLSI, 2024), *A. baumannii* is defined as CRAB if the MIC of imipenem or meropenem exceeds 8 mg/L as determined by broth microdilution. Subsequent to the revival of cryopreserved CRAB isolates, subcultures from passages two and three were subjected to broth microdilution MIC assays to evaluate susceptibility to eravacycline, polymyxin B, and additional antimicrobial agents. As neither the CLSI (CLSI, 2024) nor the European Committee on Antimicrobial Susceptibility Testing (EUCAST) (EUCAST, 2025) has established official susceptibility breakpoints for eravacycline against *A. baumannii*, susceptibility was evaluated according to the breakpoint criteria provided by the Chinese Committee on Antimicrobial Susceptibility Testing (ChiCAST) (Ecast et al., 2025). According to the ChiCAST breakpoint for eravacycline against CRAB, isolates exhibiting a MIC ≤1 mg/L were classified as susceptible. For comparative purposes, the FDA-approved breakpoint for eravacycline susceptibility in Enterobacterales (MIC ≤0.5 mg/L) (FDA, 2025) is also presented, although this criterion is not directly applicable to *A. baumannii*.

The bacterial strain was retrieved from the preservation tube, inoculated into 5 mL of sterile LB broth, and then subjected to shaking incubation at 37 °C and 220 rpm for 16 h. Genomic DNA was subsequently extracted using the Tiangen Bacterial Genomic DNA Extraction Kit (Tiangen Biotech Co., Ltd., Beijing, China) and stored at −20 °C for future analysis. Polymerase chain reaction (PCR) was used to detect resistance genes, including carbapenemase (*bla*_*KPC*_, *bla*_*IMP*_, *bla*_*VIM*_, *bla*_*GIM*_, *bla*_*NDM*_, *bla*_*SME*_, *bla*_*OXA-23*_, *bla*_*OXA-48*_, *bla*_*OXA-181*_), non-ESBL genes (*TEM-1*, *SHV*) and aminoglycosides (*armA*, *ant*3, *aac6-IB*, *rmtB*). The positive products were sequenced, and the sequencing results were compared using the basic local alignment search tool (BLAST) available at http://www.ncbi.nlm.nih.gov/BLAST.

#### 2.1.3 Treatment regimen

Eravacycline (produced by Everest Medicines, National Drug Approval Number: HJ20233135, 50 mg per vial) was administered intravenously at a dose of 1 mg/kg every 12 h, with each infusion completed within 60 min. The choice of adjunctive antimicrobial agents was guided by antimicrobial susceptibility testing results, prioritizing agents that exhibit low MICs, favorable clinical availability, and minimal toxicity profiles. The recommended duration of eravacycline therapy is 7–10 days. The treating clinician adjusted the length of treatment according to the patient’s clinical status.

#### 2.1.4 Primary and secondary endpoints

The primary endpoint of this study was the clinical response rate. Upon completion of the eravacycline-based combination therapy regimen, an assessment was performed to determine whether the patient achieved a clinical response. The resolution or improvement of symptoms, clinical signs, laboratory parameters, and imaging findings related to pulmonary infection was considered a clinical response. The clinical response rate was calculated as the proportion of patients exhibiting a clinical response relative to the total number of patients evaluated.

The secondary endpoints were the bacterial eradication rate, 28-day all-cause mortality rate, and incidence of adverse events. The total number of eradication cases was determined by summing both complete and partial eradication instances. The overall eradication rate was calculated as the proportion of these total eradication cases relative to the total number of cases studied. Eradication was defined as the absence of the initial pathogen in cultures obtained from specimens collected at the original infection site following treatment. Presumed eradication was applied in situations where clinical cure was achieved, but culture sampling was not feasible due to the resolution of disease symptoms (e.g., absence of sputum or bronchoalveolar lavage fluid) or when the sampling procedure was excessively invasive; under these circumstances, bacterial eradication was inferred. Partial eradication was characterized by a reduction in bacterial load by two grades (++) or a change in the predominance status of the pathogen. Non-eradication was defined by the continued presence of the original pathogen in cultures from specimens collected at the initial infection site after treatment. The evaluation of causality for adverse events was conducted utilizing the WHO-Uppsala Monitoring Centre (WHO-UMC) (WHO, 2013) causality assessment framework. Events were categorized as follows: certain, probable, possible, unlikely, conditional/unclassified, or unassessable. This classification was determined by considering factors such as the temporal association between drug administration and event occurrence, established patterns of drug reactions, information from dechallenge and rechallenge procedures, and the exclusion of alternative etiologies.

#### 2.1.5 Plasma concentration measurement

Sample Collection: Considering that the average half-life of eravacycline is approximately 20 h with notable interindividual variability, blood samples were obtained at steady state following the administration of the 13th dose. Venous blood specimens (2 mL) were drawn at the following time points: immediately prior to dosing (0 h) and at 1, 2, 4, 7, and 11 h post-dosing. The samples were collected in EDTA-anticoagulated tubes and centrifuged at 5000 rpm for 5 min. The resulting plasma (2 mL) was stored at −80 °C until further analysis.

Analytical Procedure: A 50 μL aliquot of plasma was combined with 150 μL of an eravacycline internal standard solution (TP-6693-046, Lot: PH-189-10A, MRIGlobal). The resulting mixture was vortexed thoroughly and then centrifuged at 5000 rpm for 5 min. Subsequently, 100 μL of the supernatant was diluted with an equal volume of water. After additional vortexing and a second centrifugation under the same conditions, a final 100 μL of the supernatant was collected for analysis. Plasma eravacycline concentrations were quantified using high-performance liquid chromatography coupled with tandem mass spectrometry (HPLC–MS/MS). Chromatographic separation was achieved with mobile phase A (water containing 0.1% formic acid) and mobile phase B (methanol containing 0.1% formic acid) at a flow rate of 0.4 mL/min, with the column maintained at 40 °C and an injection volume of 10 μL. Mass spectrometric detection was conducted using an electrospray ionization (ESI) source operating in positive ion mode with multiple reaction monitoring (MRM). Instrument parameters included an ion source voltage of 5.5 kV, ion source temperature of 450 °C, curtain gas pressure of 35 psi, nebulizing gas pressure of 40 psi, and auxiliary gas pressure of 40 psi. Detailed information regarding the analyte and internal standard ion pairs, declustering potential (DP), collision energy (CE), and retention times is provided in Table 1. This analytical method was developed and validated in accordance with the FDA’s bioanalytical method validation guidelines.

**TABLE 1:** Mass spectral parameters and retention times of eravacycline and the internal standard.

Compound	*m/z* of precursor ion	*m/z* of product ion	DP (*V*)	CE (*V*)	tR (min)
ERA	559.8	542.2	31	50	2.37
IS	567.1	550.2	130	50	2.37

ERA: eravacycline; IS: internal standard; DP: declustering potential; CE: collision energy; tR: retention time.

#### 2.1.6 Clinical data collection

Patient demographic and clinical characteristics, encompassing age, sex, preexisting medical conditions, and comorbidities, were systematically recorded. The dataset further comprised vital signs, the Clinical Pulmonary Infection Score (CPIS), the Acute Physiology and Chronic Health Evaluation II (APACHE II) score, the Sequential Organ Failure Assessment (SOFA) score, laboratory findings, pulmonary or chest computed tomography (CT) imaging results, types of respiratory support employed, respiratory specimen culture outcomes, and detailed information regarding eravacycline administration and concomitant antimicrobial therapies, including dosage and duration. Moreover, patient outcomes were assessed at 28 days.

### 2.2 Statistical analysis

Statistical analyses were performed using SPSS version 22.0. The normality of the distribution of continuous variables was assessed prior to analysis. Continuous variables demonstrating a normal distribution are reported as the mean ± standard deviation (±SD), and comparisons between groups were conducted using independent-samples *t* tests or one-way analysis of variance (ANOVA). For continuous variables that did not meet normality assumptions, data are presented as medians with interquartile ranges [M (Q1, Q3)], and between-group comparisons were conducted using the Mann–Whitney U test. Categorical variables are expressed as percentages (%), and they were analyzed using Fisher’s exact test, as appropriate. For normally distributed paired continuous variables, comparisons were performed using the paired *t*-test; for non-normally distributed paired continuous variables, comparisons were performed using the Wilcoxon signed-rank test. Statistical significance was set at a two-tailed alpha level of 0.05, and *p* values are reported alongside corresponding effect size measures. For continuous variables, effect sizes were calculated using Hedges’g to account for small sample bias with 95% confidence intervals estimated via the noncentral *t* distribution. For categorical variables, odds ratios (ORs) with 95% confidence intervals were computed using exact methods. Effect sizes for the paired *t*-test are reported using Cohen’s_dz_, with 95% confidence intervals. For the Wilcoxon signed-rank test, effect sizes are represented by the *r* statistic. Given the small sample size and skewed distributions of certain continuous variables, effect sizes were not calculated for variables that lacked normality. To investigate potential confounding variables influencing the clinical response, we employed inverse probability of treatment weighting (IPTW) as an adjustment method. This weighting approach was used to balance the distribution of covariates across groups, thereby mitigating their confounding impact on clinical response. Furthermore, point-biserial correlation coefficients were computed to investigate the relationship between binary clinical outcomes (response versus nonresponse) and continuous pharmacokinetic (PK) parameters.

## 3 Study results

### 3.1 Demographic and clinical characteristics

As shown in Table 2, this study encompassed a cohort of 22 patients. Among these patients, 21 had VAP, and one had HAP. Most were male (*n* = 14, 63.6%), with a mean age of 62.86 years (range: 52–82 years). Comorbidities were present in 77.3% (*n* = 17) of the cohort, with diabetes mellitus (36.4%) and hypertension (33.3%) being the most prevalent underlying conditions. All patients demonstrated varying degrees of organ dysfunction, predominantly respiratory failure (*n* = 17, 94.4%), followed by renal insufficiency (31.8%). Furthermore, 22.7% (*n* = 5) of the patients experienced concurrent septic shock. Regarding disease severity, the mean CPIS was 7.5, with all patients exhibiting CPIS values greater than 6. The mean SOFA score was 6.23, and the mean APACHE II score was 13.77. Regarding prior therapeutic interventions, six patients had been treated with tigecycline before the initiation of eravacycline-based combination therapy during the current hospitalization, and 11 patients had a history of glucocorticoid administration.

**TABLE 2:** Baseline characteristics.

Parameters	Total (*n* = 22)
Demographics	
Pneumonia classification, *n* (%)	
Hospital-acquired pneumonia	1 (4.5)
Ventilator-associated pneumonia	21 (95.5)
Age (years)	62.86 ± 13.04
Sex, *n* (%)	
Female	8 (36.4)
Male	14 (63.6)
Septic shock, *n* (%)	
No	17 (77.3)
Yes	5 (22.7)
Organ injury, *n* (%)	
Respiratory	21 (95.5)
Kidney	7 (31.8)
Liver	5 (22.7)
Heart	2 (9.1)
Comorbidity, *n* (%)	17 (77.3)
Diabetes	8 (36.4)
Hypertension	6 (33.3)
COPD	3 (13.6)
Bronchiectasis	4 (19.1)
Coronary heart disease	3 (13.6)
Using glucocorticoids, *n* (%)	11 (50.0)
History of tigecycline use, *n* (%)	6 (27.3)
SOFA score	6.23 ± 3.77
APACHE II score	13.77 ± 6.23
CPIS score	7.50 ± 1.10
Vital signs	
*T*_max_ (°C)	37.64 ± 0.74
HR (beats/min)	96.09 ± 18.86
MAP (mmHg)	81.33 ± 13.93
PO_2_ (mmHg)	129.50 ± 37.58
Oxygenation index	306.39 ± 87.89
Laboratory indicators	
WBC (×10^9^/L)	13.75 ± 4.23
Lymphocyte count (×10^9^/L)	0.95 ± 0.51
Platelet counts (×10^9^/L)	222.64 ± 111.58
Procalcitonin (PCT, ng/mL)	0.64 (0.30,2.06)
Total bilirubin (TBIL, μmol/L)	15.55 (6.45,24.40)
ALT (U/L)	27.50 (15.50,76.00)
AST (U/L)	33.00 (20.75,42.50)
BUN (mmol/L)	16.60 (8.25,22.75)
Creatinine (μmol/L)	79.50 (54.5,149.25)
Pathogen	
*Acinetobacter* baumannii colony count, *n* (%)	
3+	9 (40.9)
4+	13 (59.1)
Eravacycline MIC, *n* (%)	
0.125 mg/L	2 (9.1)
0.25 mg/L	6 (27.3)
0.5 mg/L	9 (40.9)
0.75 mg/L	2 (9.1)
1 mg/L	3 (13.6)

* COPD, chronic obstructive pulmonary disease; HFNC, High-Flow Nasal Cannula; HR: heart rate; MAP, Mean- Arterial Pressure; WBC, white blood cell count; PLT, Platelet-Count; ALT, alanine aminotransferase; AST, aspartate aminotransferase; BUN, blood urea nitrogen; MIC, minimum inhibitory concentration.

### 3.2 *In Vitro* drug susceptibility testing outcomes and drug resistance gene detection results for eravacycline in CRAB

As shown in Table 2, the MIC values of eravacycline, determined using the broth microdilution method, were all ≤ 1 mg/L, with most (66.7%) exhibiting MICs ≤0.5 mg/L. When categorized according to the FDA Enterobacterales susceptibility breakpoint (≤0.5 mg/L), 17 isolates (77.3%) demonstrated MICs at or below 0.5 mg/L, whereas five isolates (22.7%) exhibited MICs above 0.5 mg/L, ranging from 0.75 to 1 mg/L. Additionally, resistance gene analysis was conducted on the available isolates, identifying significant resistance determinants in 18 of the 22 isolates examined (see Table 3). The analysis demonstrated that all strains harbored the *bla*_*OXA-23*_, *TEM-1*, and *armA* resistance genes. In contrast, the *ant3* gene was present in 33.3% of isolates, and the *aac6-IB* gene was detected in 22.2%. The following resistance genes were not detected in any isolates: *bla*_*KPC*_, *bla*_*IMP*_, *bla*_*VIM*_, *bla*_*GIM*_, *bla*_*NDM*_, *bla*_*SME*_, *bla*_*SHV*_, *bla*_*OXA-48*_, *bla*_*OXA-181*_, *rmtB*.

**TABLE 3:** Eravacycline minimum inhibitory concentration (MIC) and resistance genes of the patient’s pathogenic bacteria.

Number	*bla*_*OXA-23*_	*TEM-1*	*armA*	*ant3*	*aac6-IB*
1	+	+	+	+	+
2	+	+	+	+	−
3	+	+	+	+	−
4	+	+	+	−	−
5	+	+	+	−	−
6	+	+	+	+	+
7	+	+	+	−	−
8	+	+	+	−	−
9	+	+	+	−	−
10	+	+	+	−	−
11	+	+	+	−	−
12	+	+	+	+	+
13	+	+	+	−	−
14	+	+	+	−	−
15	+	+	+	+	+
16	+	+	+	−	−
17	+	+	+	−	−
18	+	+	+	−	−
Detection rate (%)	100%	100%	100%	33.3%	22.2%

### 3.3 Eravacycline treatment and clinical outcomes

As shown in Table 4, this study included 19 patients treated with a combination of eravacycline and polymyxin B. Among these patients, 16 received eravacycline at a dosage of 1 mg/kg every 12 h, while three diagnosed with concurrent CRAB bacteremia received an elevated dose of 1.5 mg/kg every 12 h via intravenous infusion. Additionally, three patients were treated with a combination of eravacycline and cefoperazone-sulbactam, with eravacycline delivered intravenously at 1.0 mg/kg every 12 h. The average duration of eravacycline therapy was 10.95 days. Upon completion of the eravacycline treatment regimen, radiological improvement was noted in 63.6% of patients. Among the 22 patients evaluated, 16 showed clinical improvement in pulmonary infection, while six did not, yielding a clinical response rate of 72.7% (16/22). At the 28-day follow-up post-initiation of eravacycline therapy, three deaths were documented, corresponding to a 28-day all-cause mortality rate of 13.6%. Regarding microbiological efficacy, bacterial eradication was observed in 17 patients, partial eradication in two, presumed eradication in two, and no eradication in one, resulting in an overall bacterial clearance rate of 86.4%.

**TABLE 4:** Treatment details of eravacycline for patients and clinical outcomes.

Parameters		Total (*n* = 22)
Current therapy		
Antibiotic regimens, *n* (%)		
​	Eravacycline + PB	19 (86.4)
​	Eravacycline + SCF	3 (13.6)
ERA duration (Day)		10.95 ± 2.95
Vasopressor, *n* (%)		
​	No	15 (68.2)
​	NE	7 (31.8)
Ventilation type, *n* (%)		
​	Nasal cannula	5 (22.7)
​	HFNC	2 (9.1)
​	Invasive ventilation	15 (68.2)
Clinical outcomes		
CT change, *n* (%)		
​	Improve	14 (63.6)
​	Same	2 (9.1)
​	Progress	5 (22.7)
​	Not value	1 (4.5)
Micro-outcome, n (%)		
​	Eradication	17 (77.3)
​	Presumptive eradication	2 (9.1)
​	Partial eradication	2 (9.1)
​	Persistence	1 (4.5)
Post-treatment CPIS score		3.00 ± 1.76
Duration of mechanical ventilation (d)		15.50 (8.75, 29.50)
D28 outcome (%)		
​	Survive	19 (86.4)

* CT, computed tomography; ERA, eravacycline; HFNC, High-Flow Nasal Cannula; PB, Polymyxin-B; SCF, Cefoperazone/Sulbactam.

An additional analysis was conducted to evaluate clinical outcomes in relation to eravacycline MIC, combination therapy, and dosage, as presented in Table 5. To determine the influence of MIC on clinical response, patients were stratified according to the FDA Enterobacterales susceptibility breakpoint (≤0.5 mg/L versus >0.5 mg/L). Comparative analysis revealed no significant differences between the two MIC groups regarding clinical improvement rates (76.5% versus 60%, *p =* 0.585, Adjusted OR: 5.106, 95% CI: 0.266–94.419, *p =* 0.282), microbiological eradication rates (82.4% versus 80%, *p =* 0.675), or 28-day mortality rates (82.4% versus 100%, *p =* 1.000).

**TABLE 5:** Adjusted analysis of clinical outcomes according to MIC, combination regimen, and dose.

Outcomes	MIC of EVA		OR (95% CI)	*p*	Adjusted OR^a^ (95% CI)	*p*
	MIC ≤0.5 mg/L (*n* = 17)	MIC >0.5 mg/L (*n* = 5)				
Clinical improvement, *n* (%)	13 (76.5)	3 (60.0)	0.462 (0.056–3.811)	0.585	5.106 (0.266–94.419)	0.282
Microbiological eradication, *n* (%)	14 (82.4)	4 (80.0)	0.857 (0.069–10.666)	0.675	​	​
28-day mortality, *n* (%)	14 (82.4)	5 (100.0)	-	1.000	​	​

Outcomes	Combination regimen		OR (95% CI)	*p*	Adjusted OR^b^ (95% CI)	*p*
	ERA + PB (*n* = 19)	ERA + SCF (*n* = 3)				
Clinical improvement, *n* (%)	15 (78.9)	1 (33.3)	0.133 (0.009–1.872)	0.169	0.166 (0.010–2.782)	0.212
Microbiological eradication, *n* (%)	16 (84.2)	2 (66.7)	0.235 (0.014–3.917)	0.371	​	​
28-day mortality, *n* (%)	17 (89.5)	2 (66.7)	0.375 (0.025–5.572)	0.470	​	​

Outcomes	Dose		Or (95% CI)	*p*	Adjusted OR^c^ (95% CI)	*p*
	1.0 mg/kg (*n* = 19)	1.5 mg/kg (*n* = 3)				
Clinical improvement, *n* (%)	13 (76.5)	3 (100.0)	-	0.532	-	0.999
Microbiological eradication, *n* (%)	16 (84.2)	2 (66.7)	0.375 (0.025–5.572)	0.470	​	​
28-day mortality, *n* (%)	17 (89.5)	2 (66.7)	0.235 (0.15–3.917)	0.371	​	​

MIC, minimum inhibitory concentration; ERA, eravacycline; PB, Polymyxin-B; SCF, Cefoperazone/Sulbactam.

^a^ Adjusted for combination regimen, eravacycline dose, age, and SOFA, score using IPTW.

^b^ Adjusted for eravacycline dose, MIC, age, and SOFA, score using IPTW.

^c^ Adjusted for combination regimen, MIC, age, and SOFA, score using IPTW.

In the context of combination therapy, although the clinical improvement rate was higher in the eravacycline plus PB group (76.5%) than in the eravacycline plus SCF group (33.3%), this difference did not reach significance (*p* = 0.169, Adjusted OR: 0.166, 95% CI: 0010–2.782, *p =* 0.212). Additionally, microbiological eradication rates and 28-day mortality rates were similar between the two combination therapy groups (*p* = 0.470 and *p* = 0.371). In the subgroup analysis by dosage, the higher-dose group (1.5 mg/kg) exhibited a significantly higher microbiological eradication rate than the standard-dose group. However, no significant differences were observed in clinical response (*p* = 0.532), the microbiological eradication rate (*p* = 0.470), or 28-day mortality (*p* = 0.371) between the two dosage groups.

### 3.4 Incidence of adverse reactions

**FIGURE 1:**
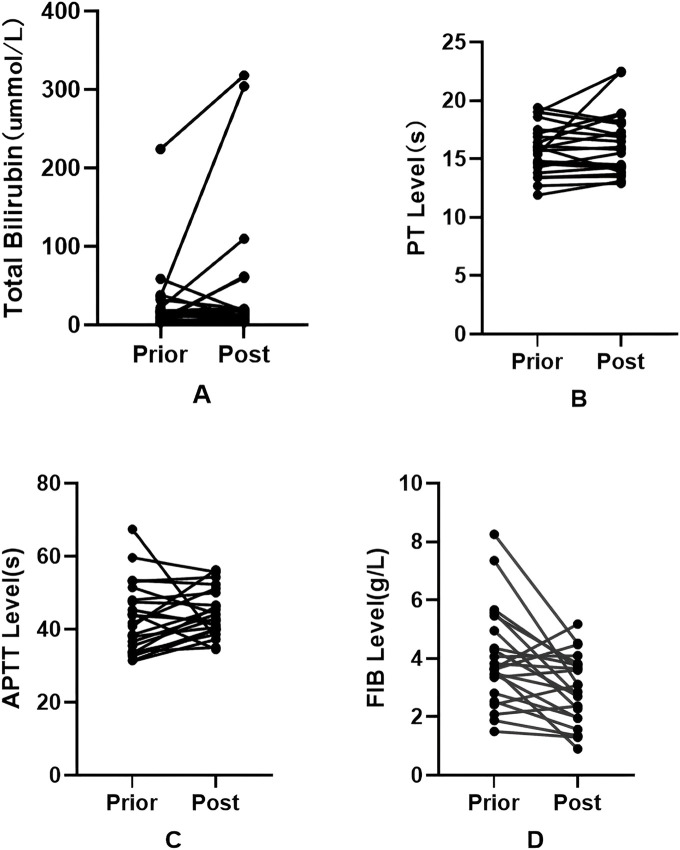
Changes in patients' total bilirubin levels, prothrombin time (PT), activated partial thromboplastin time (APTT), and fibrinogen (FIB) levels.

Throughout the treatment period, no common adverse events, such as infusion reactions, allergic responses, vasculitis, nausea, or vomiting, were observed. Pertinent laboratory parameters were meticulously monitored to evaluate potential eravacycline-associated disturbances in bilirubin metabolism and coagulation function (see Table 6; Figure 1). The comparative analysis of serum total bilirubin, prothrombin time (PT), activated partial thromboplastin time (APTT), and fibrinogen (FIB) levels before and after treatment revealed no significant differences for total bilirubin (*p* = 0.314), PT (*p* = 0.366), or APTT (*p* = 0.127). However, a significant decrease in fibrinogen concentration was detected post-treatment (4.00 ± 1.68 g/L pre-treatment vs. 2.96 ± 1.16 g/L post-treatment; *p* = 0.040, Cohen’s_dz_ = 0.699). Within the study cohort, five patients developed hypofibrinogenemia (defined as FIB <2.0 g/L), including two cases of severe hypofibrinogenemia (FIB <1.5 g/L). Notably, neither patient with severe hypofibrinogenemia experienced clinically significant bleeding complications, such as gastrointestinal hemorrhage, hemoptysis, or intracranial bleeding, and no transfusions of blood products were necessary.

**TABLE 6:** Changes in patients’ total bilirubin levels, prothrombin time (PT), activated partial thromboplastin time (APTT), and fibrinogen (FIB) levels.

Parameters	Prior treatment	Post treatment	Effect size*	*p*
TB (μmol/L)	15.55 (6.45, 24.40)	13.90 (7.50, 31.48)	0.215	0.314
PT (s)	15.80 (14.18, 17.25)	15.80 (14.05, 18.10)	0.193	0.366
APTT (s)	41.25 (33.83, 48.85)	43.50 (39.58, 50.30)	0.325	0.127
FIB (g/L)	4.00 ± 1.68	2.96 ± 1.16	0.699	0.04

* Effect sizes for the paired t-test are reported using Cohen’s_dz_, with 95% confidence intervals; effect sizes for the Wilcoxon signed-rank test are represented by the *r* statistic.

### 3.5 Comparison of patients with and without clinical failure

Table 7 presents a comparative analysis of the baseline characteristics and treatment outcomes of patients who achieved clinical response and those who did not (clinical failure). The findings indicate no significant differences between the two groups regarding age, sex, comorbidities, organ function, bacterial isolate counts, eravacycline MIC, pre-treatment vital signs, laboratory values, SOFA scores, or APACHE II scores. Notably, the oxygenation index in the clinical response group prior to treatment was significantly higher than that in the clinical failure group (*p* < 0.05). Following administration of the eravacycline-based combination therapy, the failure group demonstrated significantly elevated SOFA and APACHE II scores, peak body temperature (T_max_), and blood urea nitrogen levels compared with the response group, with all differences reaching significance (*p* < 0.05).

**TABLE 7:** Comparison between patients with and without clinical failure.

Parameters	Clinical failure	Clinical response	Effect size*	*p*
	(*n* = 6)	(*n* = 16)		
Demographics				
Age (years)	69.67 ± 10.05	60.31 ± 13.38	0.712 (−0.255–1.633)	0.138
Sex, *n* (%)	​	​	1.200 (0.166–8.659)	1.000
Female	2 (33.3)	6 (37.5)	​	​
Male	4 (66.7)	10 (62.5)	​	​
Septic shock, *n* (%)	​	​	1.667 (0.147–18.874)	1.000
No	5 (83.3)	12 (75.0)	​	​
Yes	1 (16.7)	4 (25.0)	​	​
Organ injury, *n* (%)				
Respiratory	6 (100.0)	15 (93.8)	1.067 (0.940–1.211)	1.000
Kidney	1 (16.7)	6 (37.5)	3.000 (0.279–32.209)	0.616
Liver	0 (0.0)	5 (31.3)	-	0.266
Heart	2 (33.3)	0 (0.0)	-	0.065
Comorbidity, *n* (%)				
Diabetes	2 (33.3)	6 (37.5)	1.200 (0.166–8.659)	1.000
Hypertension	2 (33.3)	5 (31.3)	0.909 (0.123–6.715)	1.000
COPD	0 (0.0)	3 (18.8)	1.231 (0.973–1.557)	0.532
Bronchiectasis	0 (0.0)	1 (6.3)	0.286 (0.030–2.719)	0.407
Coronary heart disease	2 (33.3)	1 (6.3)	0.133 (0.009–1.872)	0.169
Pathogen				
Eravacycline MIC, *n* (%)	​	​	-	0.095
0.125 mg/L	0 (0.0)	2 (12.5)	​	​
0.25 mg/L	0 (0.0)	6 (37.5)	​	​
0.5 mg/L	4 (66.6)	5 (31.25)	​	​
0.75 mg/L	1 (16.7)	1 (6.25)	​	​
1.00 mg/L	1 (16.7)	2 (12.5)	​	​
*Acinetobacter baumannii* colony count, *n* (%)	​	​	1.667 (0.251–11.071)	0.655
3+	3 (50.0)	6 (37.5)	​	​
4+	3 (50.0)	10 (62.5)	​	​
History of tigecycline use, *n* (%)	2 (33.3)	4 (25.0)	0.667 (0.087–5.127)	1.00
Using immunosuppressant, *n* (%)	4 (66.7)	7 (43.8)	0.389 (0.055–2.771)	0.635
Pre-treatment parameters with ERA				
SOFA score	7.0 (3.5, 11.8)	6.0 (3.0, 7.00)	-	0.347
APACHE II score	15.17 ± 4.67	13.25 ± 6.78	0.292 (−0.619–1.195)	0.456
Pre-treatment CPIS score	7.50 ± 0.54	7.5 ± 1.26	0.00 (−0.938–0.938)	1.000
Vital signs				
*T*_max_ (°C)	37.40 ± 0.80	37.73 ± 0.72	−0.430 (−1.337–0.487)	0.914
HR (beats/min)	93.17 ± 24.92	97.19 ± 16.91	−0.238 (−1.103–0.706)	0.667
MAP (mmHg)	88.06 ± 11.83	78.81 ± 14.15	0.653 (−0.279–1.570)	0.171
Oxygenation index	277.22 (218.68, 360.83)	356.00 (244.17, 416.46)	-	0.024
Laboratory indicators				
WBC (×10^9^/L)	14.44 ± 5.59	13.49 ± 3.79	0.252 (−0.695–1.190)	0.649
PLT (×10^9^/L)	170.00 ± 104.55	242.38 ± 110.74	−0.637 (−1.553–6.294)	0.182
PCT (ng/mL)	0.4585 (0.2833, 3.4575)	0.699 (0.304, 1.53)	-	0.802
Total bilirubin (TBIL, μmol/L)	13.75 (8.325, 22.375)	16.25 (5.75, 29.6)	-	0.483
ALT (U/L)	32 (10.25, 55.75)	27.5 (19.75, 109.75)	-	0.396
AST (U/L)	27.5 (18.5, 32)	34.5 (22.25, 57.5)	-	0.110
Blood urea nitrogen (BUN, mmol/L)	20.55 (10.075, 29.8)	13.4 (8.275, 20.4)	-	0.302
Creatinine (μmol/L)	67.5 (52.75,232)	83 (67.5,134.5)	-	0.816
Post-treatment parameters with ERA				
SOFA score	6.5 (4.5,18.75)	2.0 (1.50,6)	-	0.030
APACHE II score	19.00 ± 4.69	9.31 ± 6.01	1.633 (0.584–2.650)	0.002
Post-treatment CPIS score	4.33 ± 2.07	2.41 ± 1.36	1.138 (0.148–2.103)	0.024
Vital signs				
*T*_max_ (°C)	37.92 ± 0.49	37.09 ± 0.74	1.167 (0.183–2.126)	0.020
HR (beats/min)	93.00 ± 14.91	90.81 ± 12.71	0.158 (−0.748–1.060)	0.735
MAP (mmHg)	81.28 ± 16.46	83.08 ± 15.89	−1.108 (−1.010–0.796)	0.758
Oxygenation index	279.27 ± 100.05	337.57 ± 90.92	−0.601 (−1.515–0.327)	0.207
Laboratory indicators				
WBC (×10^9^/L)	15.45 ± 6.52	10.61 ± 5.23	0.833 (−0.115–1.762)	0.086
PLT (×10^9^/L)	131.83 ± 102.54	256.81 ± 140.75	−0.909 (−1.844–0.046)	0.062
PCT (ng/mL)	1.1565 (0.3443, 6.765)	0.259 (0.196, 0.6288)	-	0.083
Total bilirubin (TBIL, μmol/L)	16.5 (10.525, 92.425)	12.45 (6.625, 50.275)	-	0.483
ALT (U/L)	33.00 ± 23.87	65.00 ± 68.16	−0.511 (−1.421–0.411)	0.280
AST (U/L)	68.33 ± 68.51	56.69 ± 40.57	0.228 (−0.680–1.131)	0.625
Blood urea nitrogen (BUN, mmol/L)	29.6 (17.85, 63.35)	11.65 (7.43, 19.18)	-	0.011
Creatinine (μmol/L)	182 (89.25, 399.25)	101 (43.75, 182)	-	0.134

* COPD, chronic obstructive pulmonary disease; HFNC, High-Flow Nasal Cannula; ERA, eravacycline; PB, Polymyxin-B; SCF, Cefoperazone/Sulbactam; HR, heart rate; MAP, Mean-Arterial Pressure; WBC, white blood cell count; PLT, Platelet-Count; ALT, alanine aminotransferase; AST, aspartate aminotransferase; BUN, blood urea nitrogen.

* For continuous variables, effect sizes were calculated using Hedges’ g to account for small sample bias with 95% confidence intervals estimated via the noncentral *t* distribution. For categorical variables, odds ratios (ORs) with 95% confidence intervals were computed using exact methods. Given the small sample size and the skewed distribution of certain continuous variables, effect sizes were not calculated for those variables that lacked normality.

### 3.6 Eravacycline plasma concentration monitoring results

**FIGURE 2:**
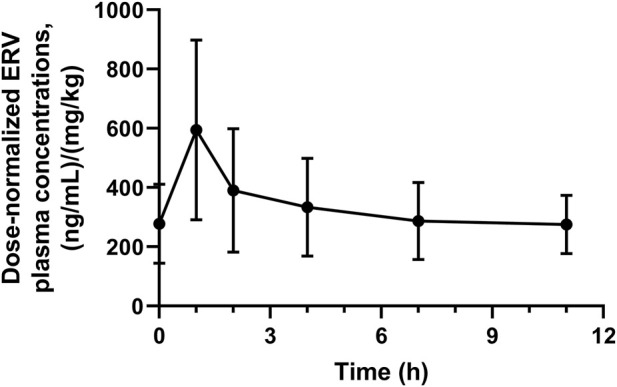
The curve diagram of the standardized average plasma concentration over time of intravenous infusion of eravacycline in patients.

Plasma concentration monitoring of eravacycline was completed in a cohort of 15 patients. Among these patients, 13 received eravacycline at 1.0 mg/kg every 12 h, while two received 1.5 mg/kg every 12 h. One patient’s data was excluded from the statistical analysis due to sampling errors. The distribution of dose-normalized plasma concentrations is presented in Figure 2.

Following a 60-min intravenous infusion, plasma concentrations rapidly reached peak levels and then declined rapidly. At 4 h post-infusion, the rate of decline markedly decelerated, and concentrations gradually stabilized. Pharmacokinetic parameter analysis revealed considerable interindividual variability among patients, with coefficients of variation ranging from 40.53% to 86.32% (Table 8).

**TABLE 8:** Parameters of patients receiving intravenous infusion of eravacycline.

Patient	AUC_ss_	AUC ( 0–∞ )	t_1/2z_	*V*_z_	CL_z_	C_max_	C_min_	C_av_	DF
	(ng/mL*h)	(ng/mL*h)	(h)	(L)	(L/h)	(ng/mL)	(ng/mL)	(ng/mL)	
1	2591	11107	21	138	5	334.54	245.65	215.88	0.41
2	4176	13293	23	247	8	740.28	310.98	347.98	1.23
3	2054	3349	8	344	30	280.69	170.28	171.15	0.65
4	4622	14460	20	153	5	550.47	368.32	385.19	0.47
5	4260	20057	33	143	3	474.07	339.50	354.96	0.38
7	4586	10497	12	96	6	640.61	405.98	382.14	0.61
8	4068	12429	23	161	5	1110.52	234.99	338.99	2.58
9	2515	4833	10	155	10	304.31	181.82	209.60	0.58
10	3787	7447	12	160	9	799.50	241.68	315.56	1.77
11	4502	12027	17	125	5	799.43	294.23	375.15	1.35
12	8300	13377	8	60	5	1222.60	650.96	691.69	0.83
13	3222	12326	26	152	4	656.12	219.40	268.52	1.63
14	3069	15215	33	185	4	439.35	163.46	255.77	1.08
15	2267	3821	8	156	13	311.30	226.28	188.95	0.45
Mean	3858.50	11017.00	18.14	162.50	8.00	618.84	289.54	321.54	1.00
SD	1563.85	4711.05	8.83	66.92	6.91	295.44	127.46	130.32	0.65
CV%	40.53	42.76	48.67	41.18	86.32	47.74	44.02	40.53	64.74

(*n* = 14).

AUC, area under the curve; t1/2z: Half-life; *V*z: Apparent volume of distribution; CLz, Clearance; C_max_, Peak concentration; C_min_, trough concentration; Cav, average steady-state concentration; DF, degree of fluctuation; SD, standard deviation; CV, coefficient of variation.

* Patient 6 was excluded from the final outcome analysis due to outlier values, which attributed to an error in blood sample timing.

**FIGURE 3:**
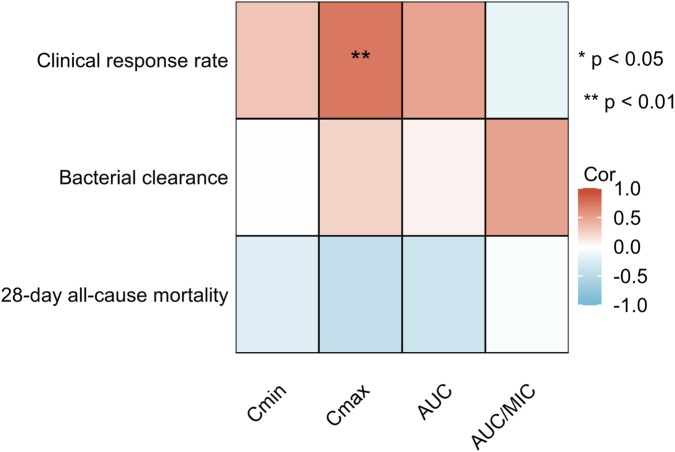
Correlation analysis between pharmacokinetic parameters of eravacycline and endpoint indicators.

**FIGURE 4:**
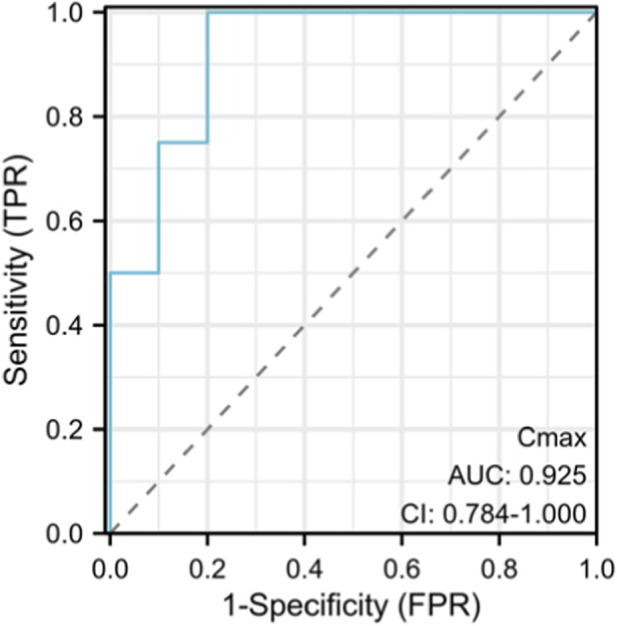
Analysis of the receiver operating characteristic curve (ROC) of the predictive power of clinical remission rate.

Subsequent correlation analyses were performed to assess the relationships between the minimum concentration (C_min_), maximum concentration (C_max_), area under the curve (AUC), and AUC to MIC ratio (AUC/MIC) with primary (clinical response rate) and secondary (bacterial eradication rate and 28-day all-cause mortality) endpoints, as illustrated in Figure 3. Exploratory receiver operating characteristic (ROC) analysis suggested that C_max_ may have potential predictive value for clinical response (AUC = 0.925; 95% CI: 0.784–1.000). However, due to the limited number of patients, this finding should be interpreted with caution and is considered hypothesis-generating (Figure 4).

## 4 Discussion

*A. baumannii* is one of the most prevalent, virulent, and problematic pathogens implicated in nosocomial infections, notable for its extensive antimicrobial resistance and high clonal transmissibility. CRAB is resistant to most currently available antibiotic therapies. The latest guidelines issued by the IDSA recommend sulbactam-durlobactam as a preferred therapeutic option for CRAB infections, advising its use in combination with either imipenem-cilastatin or meropenem (Tamma et al., 2024). Durlobactam is a β-lactamase inhibitor with strong inhibitory activity against class A, C, and D β-lactamases; however, it is ineffective against class B metallo-*β*-lactamases. Despite its efficacy, the combination of sulbactam and durlobactam is largely unavailable in most hospitals across China. The Chinese expert consensus recommends that infections caused by extensively drug-resistant *A. baumannii* are generally managed with combination therapy involving two or three antimicrobial agents. Commonly utilized two-drug regimens include combinations centered on sulbactam or sulbactam-containing compounds, tigecycline-based therapies, or polymyxin-based treatments (Zhou et al., 2014). These three categories of agents may be used in combination or alongside additional drugs with lower MIC values, as determined by susceptibility testing.

Due to tigecycline’s pharmacokinetic and pharmacodynamic properties, its concentration in alveolar lining fluid is low, limiting its efficacy in treating VAP. Multiple studies have shown that the standard dose of tigecycline results in higher mortality rates when treating CRAB pneumonia compared with polymyxin and sulbactam regimens (Iovleva et al., 2025). The overall clinical outcomes and microbiological eradication rates associated with tigecycline treatment are comparatively modest. For example, in one study, the microbiological response rate was only 48% in tigecycline-treated patients with HAP caused by multidrug-resistant *A. baumannii* (Zhou et al., 2019). Furthermore, an independent study examining antibiotic treatment strategies for critically ill patients with pneumonia caused by CRAB revealed that tigecycline-based therapy was associated with higher intensive care unit (ICU) mortality and increased incidence of treatment failure relative to non-tigecycline regimens (Liang et al., 2018). These findings indicate that although tigecycline is a potential therapeutic option, its effectiveness is likely constrained.

The effectiveness of combination regimens containing tigecycline and colistin remains limited. Kim et al. demonstrated that treatment protocols involving tigecycline and colistin achieve similar clinical success rates (47% and 48%, respectively). Furthermore, the use of combination therapy did not consistently yield superior survival outcomes relative to those achieved with colistin monotherapy (Kim et al., 2016). A 2018 randomized study by Makris and colleagues found that combination therapy (colistin with high-dose ampicillin-sulbactam and inhaled colistin) led to significantly higher rates of 5-day clinical improvement compared to colistin monotherapy (15.8% vs. 70%, *p* = 0.01). However, there was no significant difference in 28-day mortality between the two groups (63% in the monotherapy group vs. 50% in the combination group) (Makris et al., 2018). Chang et al. conducted a multicenter retrospective cohort study (n = 364) comparing polymyxin B monotherapy, tigecycline monotherapy, and their combination for hospital-acquired pneumonia caused by CRAB or CRE. The 28-day mortality was highest in the combination group (48.9%) and lowest in the polymyxin B monotherapy group (28.3%, *p* = 0.014). Multivariable Cox regression showed that polymyxin B monotherapy was independently associated with a lower risk of 28-day mortality compared with combination therapy (HR = 0.50, 95% CI: 0.31–0.81, *p* = 0.004). In the CRAB infection subgroup (n = 247), the difference in mortality between combination therapy and polymyxin B monotherapy was not statistically significant (HR = 0.61, 95% CI: 0.34–1.09, *p* = 0.093). The authors concluded that polymyxin B combined with tigecycline was not superior to polymyxin B alone and might even be associated with higher mortality (Chang et al., 2022).

The 2024 updated IDSA guidelines recommend eravacycline as a potential component of combination therapy for CRAB pneumonia; however, they emphasize that additional clinical data are necessary to support its routine use (Tamma et al., 2024). Eravacycline has garnered increasing interest due to its favorable efficacy and safety profile in the treatment of multidrug-resistant intra-abdominal infections (Lu et al., 2024). Furthermore, its application has become increasingly common in the treatment of multidrug-resistant pneumonia, especially infections caused by CRAB, demonstrating favorable therapeutic outcomes. In a case series involving 25 patients with CRAB pneumonia treated with a combination of eravacycline and ampicillin-tazobactam, 72% achieved clinical cure and 72% attained microbiological eradication (Jackson et al., 2024). Jia Jianchao et al. evaluated eravacycline in CRAB pneumonia and found that eravacycline combined with cefoperazone-sulbactam achieved a higher overall response rate (90%) than polymyxin combined with cefoperazone-sulbactam, as well as shorter duration of mechanical ventilation and lower mortality (Jia et al., 2024).

In this study, the overall clinical response rate was 72.7%, and the bacterial clearance rate was 86.4%. Although the clinical response rate observed in this study was marginally lower than that reported in previous eravacycline studies, combination regimens incorporating eravacycline demonstrated superior efficacy in treating CRAB pneumonia compared with those based on tigecycline or polymyxins. This finding suggests that eravacycline-based therapies represent a promising option for clinical application. The study population of this study exhibited substantial disease severity, as reflected by a mean SOFA score of 6.23 and an APACHE II score of 13.77, along with universal organ failure and complex comorbidities—factors well-established as predictors of poor clinical outcomes. There were certain other confounding factors that might potentially have an impact on the results. Despite the limited sample size, we have made efforts to assess those effects.

Regarding the susceptibility profile, in January 2024, the ChiCAST of the National Health Commission defined the MIC breakpoint for eravacycline against *A. baumannii* as 1 = mg/L (Ecast et al., 2025). We adopted this MIC breakpoint in this study. Nevertheless, the breakpoint for Enterobacterales established by the FDA is ≤0.5 mg/L (FDA, 2025), and the antimicrobial activity of eravacycline against *A. baumannii* observed in this study may be interpreted differently under alternative breakpoint criteria. However, in our subsequent analysis of clinical outcomes stratified by MIC, no significant differences were identified when using the ≤0.5 mg/L cutoff value (Table 5). This could be partly attributed to the small sample size in the higher MIC group (*n* = 5), which limited statistical power. More importantly, the 0.5 mg/L cutoff is derived from the FDA Enterobacterales breakpoint and may not be optimally applicable to *A. baumannii*. Thus, the selected threshold may not be the most clinically relevant discriminator for this pathogen, potentially accounting for the lack of observed difference between groups. Clinical outcomes are more influenced by host factors, infection severity, and effective combination therapy than by MIC increments.

The heterogeneity of combination regimens and dosages serves as a crucial source of confounding. To clarify this issue as comprehensively as possible, we conducted a further analysis of clinical outcomes based on combination regimens and dosages, and IPTW was employed to balance key covariates (Table 5). However, no significant differences were observed in terms of clinical response, microbiological eradication, or 28-day survival between the different combination regimen groups or between the different dose groups. The cefoperazone-sulbactam combination group consisted of only three patients, compared with 19 patients in the polymyxin B combination group. This significant imbalance not only results in extremely low statistical power but also makes any inter-group comparisons (including adjusted odds ratios) highly unstable. More importantly, the heterogeneity of combination regimens introduces a form of confounding that cannot be fully eliminated through statistical methods. Polymyxin B itself exhibits potent intrinsic activity against CRAB, while cefoperazone-sulbactam may exert synergistic effects in certain isolates. These mechanistic disparities make it difficult to isolate the net contribution of eravacycline. Even with IPTW adjustment for measured confounders, unmeasured confounding, such as clinician bias in regimen selection based on perceived illness severity or differences in treatment intensity, may still impact the results. Therefore, the subgroup analyses presented in this study should be interpreted as exploratory descriptive findings rather than comparative effectiveness conclusions. Future studies with larger sample sizes, more balanced group distributions, or randomized designs are needed to reliably evaluate the comparative value of different combination regimens in the treatment of CRAB pneumonia.

The most fundamental limitation of this study is the absence of a concurrent control group. All patients received eravacycline-based combination therapy, and the majority (86.4%) were treated with concomitant polymyxin B—an agent with potent intrinsic activity against CRAB. Consequently, the specific contribution of eravacycline versus polymyxin B cannot be isolated, the clinical response rate of 78.9% observed in the eravacycline plus polymyxin B group may reflect synergistic or additive effects of the combination rather than the independent contribution of eravacycline. This limitation is inherent to real-world observational studies, but it must be explicitly acknowledged: the findings of this study should be interpreted as an association between eravacycline-based combination regimens and favorable outcomes in a specific patient population, rather than as a causal demonstration of eravacycline efficacy. Future randomized controlled trials or studies employing more robust causal inference methods (e.g., instrumental variable analysis) are needed to elucidate the independent role of eravacycline in the treatment of CRAB pneumonia. Notably, while our findings are promising, they should be weighed against the current cautious international perspective regarding the routine use of eravacycline for CRAB pneumonia, as reflected in recent IDSA guidelines (Tamma et al., 2024) and reports of variable real-world efficacy (Kubin et al., 2025). Larger, multicenter studies are urgently needed to confirm these findings.

Numerous real-world studies and published clinical trials have identified gastrointestinal symptoms, including nausea and skin rashes, as the most frequently observed adverse reactions to eravacycline (Alosaimy et al., 2020b; Van Hise et al., 2020). Previous clinical investigations of eravacycline have established its generally well-tolerated safety profile (Solomkin et al., 2017; Solomkin et al., 2019). In a study conducted by Xiong Chunyan et al., adverse events (AEs) associated with tetracycline-class antibiotics were analyzed using data from the FDA Adverse Event Reporting System (FAERS) database (Xiong et al., 2025). This analysis revealed that among the AEs related to eravacycline, there were seven reported cases of decreased fibrinogen levels and three cases of elevated pancreatic enzyme levels.

In the current investigation, eravacycline demonstrated a generally favorable tolerability profile; no gastrointestinal adverse effects, infusion-related reactions, or vasculitis were detected. Nevertheless, we observed a significant reduction in fibrinogen concentrations during the course of treatment (*p* = 0.040). Utilizing the WHO-UMC causality assessment framework, two patients (one with FIB <1.5 g/L and one with FIB <2.0 g/L) were classified as having probable ARDs attributable to eravacycline, based on the temporal correlation with drug administration, the exclusion of alternative etiologies such as disseminated intravascular coagulation or severe hepatic dysfunction, and the established class effect of tetracycline antibiotics on fibrinogen levels. Notably, neither patient exhibiting severe hypofibrinogenemia experienced clinically significant hemorrhagic events, and neither required transfusion of blood products. Fibrinogen concentrations normalized following cessation of eravacycline therapy in both cases.

This finding has clinical significance, as hypofibrinogenemia is a well-established adverse effect associated with tigecycline, and it has also been reported as an adverse reaction related to eravacycline (Xiong et al., 2025). Although no bleeding episodes were observed in our patient cohort, the observed reduction in fibrinogen levels necessitates careful clinical consideration. The underlying mechanism may involve disruption of fibrinogen synthesis or enhanced clearance, analogous to the mechanism suggested for tigecycline. Given the extensive use of eravacycline in critically ill patients who may already present with coagulation abnormalities, we advocate for routine monitoring of fibrinogen concentrations throughout the course of therapy, especially in individuals undergoing prolonged treatment or those with preexisting coagulopathies. Further large-scale studies are required to validate this observation and to identify risk factors associated with clinically significant bleeding.

Plasma concentrations of eravacycline were assessed in a cohort of 14 patients. PK data pertaining to eravacycline in critically ill populations remain scarce. Compared with PK parameters documented in healthy individuals, both the maximum concentration (C_max_) and the area under the concentration-time curve (AUC) of eravacycline were significantly reduced in critically ill patients (C_max_: *p* = 0.004; AUC: *p* = 0.004). In contrast, the minimum concentration (C_min_), clearance (CL), and volume of distribution (*V*) did not differ significantly between the two groups. These results indicate that critically ill patients may be susceptible to subtherapeutic exposure to eravacycline.

The aforementioned phenomenon may be explained by the unique physiological and pathological characteristics observed in critically ill patients. These individuals often exhibit hypoalbuminemia, and as eravacycline demonstrates a high degree of protein binding (79%–90%), decreased albumin concentrations could result in an increased fraction of unbound drug, thereby reducing overall drug exposure. Additionally, factors such as fluid shifts in mechanically ventilated patients and organ dysfunction, including hepatic or renal impairment, may further influence the pharmacokinetics of eravacycline, complicating the optimization of dosing regimens. Consequently, therapeutic drug monitoring informed by individualized, model-based precision dosing approaches is crucial to ensure sufficient antimicrobial exposure, which is vital for enhancing clinical outcomes and minimizing the risk of toxicity. Furthermore, Ji et al. analyzed Phase I pharmacokinetic data and a lung penetration model for eravacycline, identifying an *in vitro* MIC90 of 0.5 mg/L against *A. baumannii* and a target-free drug area under the concentration-time curve to MIC ratio (fAUC/MIC) of 10 (Ji et al., 2025). However, it is critical to note that this target is derived from preclinical *in vitro* data, and it has not been prospectively validated in critically ill patients with CRAB pneumonia. In the current study, only 3 out of 15 patients (20%) achieved an fAUC/MIC ratio ≥10, and the remaining 12 patients (80%) had exposures below this threshold. The mean fAUC/MIC ratio was 9.6 ± 8.1. This high rate of target non-attainment may be explained by several factors. First, the PK/PD target derived from healthy volunteer data may not be directly translatable to critically ill patients, who often exhibit increased volume of distribution, hypoalbuminemia, and altered drug clearance. Second, achieving effective concentrations at the site of infection (epithelial lining fluid) may require higher systemic exposures than those predicted by plasma-based *in vitro* models. Additional research is necessary to integrate population PK modeling with measurements of both free drug and epithelial lining fluid concentrations to establish a robust, clinically validated PK/PD target for eravacycline in the treatment of CRAB pneumonia. Until such data are available, the present results should be considered hypothesis-generating.

An unexpected finding of this study was that C_max_, rather than AUC/MIC, emerged as the PK parameter most strongly associated with clinical response. Tetracycline-class antibiotics, including eravacycline, are typically considered AUC/MIC-driven agents according to preclinical model data. Our observation may be explained by several factors. First, in critically ill patients with pneumonia, achieving a sufficiently high peak concentration may be critical for adequate penetration into the epithelial lining fluid (ELF), the primary site of infection. Previous studies have indicated that eravacycline exhibits substantial intrapulmonary penetration, with ELF exposure exceeding free plasma exposure and peak ELF concentrations occurring early after infusion. The ELF AUC was comparable to or greater than plasma free-drug exposure, with a reported penetration ratio of approximately 6.4 (Connors et al., 2014). These data suggest that early plasma peak concentrations may help achieve adequate ELF exposure. In the setting of severe pneumonia, where pulmonary permeability and drug distribution may be altered, achieving a sufficiently high peak plasma concentration could potentially facilitate adequate lung tissue penetration. In this context, C_max_ may reflect a threshold phenomenon related to pulmonary distribution rather than a shift in the fundamental PK/PD driver. Second, the high protein binding of eravacycline complicates the interpretation of total plasma concentrations. In critically ill patients with frequent hypoalbuminemia, the unbound fraction may be increased and highly variable. It is plausible that the unbound peak concentration, rather than total exposure, is the more pharmacodynamically relevant driver of efficacy. Unfortunately, ELF concentrations were not measured in this study, which limits our ability to confirm this hypothesis. Future studies incorporating ELF sampling are needed to clarify the optimal PK/PD driver for eravacycline in pneumonia. Third, the small sample size and limited number of nonresponders may have favored the detection of C_max_ as the dominant driver, whereas AUC/MIC effects might be obscured by interindividual variability. Given these considerations, our finding that C_max_ correlates with response should be interpreted as hypothesis-generating rather than definitive. It does not negate the potential importance of AUC/MIC but rather highlights the complexity of exposure-response relationships in heterogeneous critically ill populations. Future studies should integrate population PK modeling with ELF and free drug concentration measurements to rigorously identify the optimal PK/PD index and target for eravacycline in CRAB pneumonia.

This study offers preliminary insights into the clinical utility of eravacycline-based combination therapies for treating CRAB pulmonary infections; however, several limitations must be recognized. First, as a single-center observational study with a small sample size, the findings are vulnerable to selection and measurement biases. The limited number of participants, particularly within the PK substudy, constrained the statistical power available for subgroup analyses and ROC evaluations. Moreover, multivariable adjustment for potential confounding factors, such as disease severity, organ dysfunction, and oxygenation status, was not feasible. These factors may independently affect both drug exposure and clinical outcomes. Their exclusion from adjusted analyses prevents the establishment of definitive conclusions regarding the independent association with the response. Although IPTW was employed to balance key covariates, residual confounding by unmeasured variables remains a potential issue. Additionally, this method may be unstable when dealing with small sample sizes. Thus, results from subgroup analyses with very low numbers (e.g., *n* = 3) should be interpreted with particular caution. Consequently, all *p* values and predictive performance metrics should be regarded as exploratory. Second, the lack of a concurrent control group precludes the definitive attribution of observed clinical outcomes to the treatment regimen and limits the ability to quantify its comparative advantages over alternative therapies. Third, heterogeneity arising from variability in the selection of concomitant antimicrobial agents—although reflective of real-world clinical practice−complicates the precise assessment of the individual contribution of eravacycline or specific combination regimens. Fourthly, the clinical response was evaluated by the treating clinicians without blinding, which might introduce assessment bias, especially for subjective elements. To alleviate this issue, the response was defined through a combination of objective and semi-objective criteria, and all cases were reviewed by at least two investigators to guarantee consistency. However, the lack of independent adjudication still persists as a limitation. Finally, the 28−day follow−up period may be insufficient to assess long-term outcomes. Therefore, future large-scale, multicenter randomized controlled trials are warranted to corroborate these findings and to elucidate optimal companion drugs, treatment durations, and individualized dosing strategies informed by therapeutic drug monitoring.

## 5 Conclusion

In this study, we systematically ascertained the MIC of eravacycline against CRAB, analyzed the resistance genes of CRAB, investigated its clinical response and safety, and conducted blood concentration monitoring and PK analysis. The preliminary findings suggest that the isolated CRAB strains demonstrated favorable *in vitro* susceptibility to eravacycline (ChiCAST breakpoint ≤1 mg/L). Individualized eravacycline-based combination regimens were associated with favorable clinical and microbiological outcomes, with patients demonstrating favorable tolerability and a low incidence of adverse reactions. Additionally, the initially measured blood concentration data offer a reference for exploring its PK/PD targets. Nevertheless, considering the single-center, small-sample, and observational nature of this study, these results should be regarded as preliminary. In particular, subgroup comparisons and PK/PD analyses are exploratory and necessitate validation in larger prospective cohorts.

## Data Availability

The original contributions presented in the study are included in the article/supplementary material, further inquiries can be directed to the corresponding authors.
